# Extracorporeal Membrane Oxygenation (ECMO) and hospital economics

**DOI:** 10.1051/ject/2025043

**Published:** 2025-12-17

**Authors:** Anna Ratiu, Julie A. Linick, Angelica Oyugi, Jennifer Williams, Kael Wherry, Eric Klavetter

**Affiliations:** 1 Medtronic, plc Mounds View MN USA

**Keywords:** Extracorporeal membrane oxygenation (ECMO), Payment, Costs, Length of stay, Venovenous (VV) ECMO, Venoarterial (VA) ECMO, Central ECMO, Continuous ECMO

## Abstract

*Background*: Extracorporeal membrane oxygenation (ECMO) is a powerful, life-prolonging technology, but is resource-intensive. *Methods*: This retrospective analysis utilized Medicare Fee-for-Service (FFS) inpatient data (October 2019–December 2022) to identify ECMO hospitalizations with continuous FFS coverage. We assessed total payment, costs, length of stay (LOS), and predominant medical severity diagnosis-related group (MS-DRG) and International Classification of Diseases (ICD-10) codes. Outcomes were stratified by DRG code, ICD-10 procedure codes, and ECMO type. Hospital costs were estimated using cost-to-charge ratios from the Centers for Medicare and Medicaid Services with a 3-year lag. *Results*: Continuous ECMO within MS-DRG 003 discharged between January 1, 2020, and December 31, 2022, were a subset of hospitalizations analyzed. For continuous central (CC) ECMO, mean costs were $287,334, median costs were $223,766, and the standard deviation (SD) was $224,261. Mean payment was $264,021, median was $218,642, and SD was $157,882. For continuous venoarterial (VA) ECMO, mean costs were $245,448, median was $191,363, and SD was $212,822. Mean payment was $241,617, median was $202,199, and SD was $145,456. For continuous venovenous (VV) ECMO, mean costs were $329,111, median was $256,706, and SD was $285,745. Mean payment was $298,141, median was $237,679, and SD was $198,160. The majority of patients (64%) received VA, which was the only ECMO type with a median payment that surpassed median costs. Rural facilities and teaching hospitals had consistently higher mean frequencies among all ECMO claims (CC, VA, VV) from October 2019 to December 2022 compared to their counterparts (Rural: CC = 2.4, VA = 9.3, VV = 4.9; Urban: CC = 1.4, VA = 6.1, VV = 2.2; Teaching: CC = 2.1, VA = 8.6, VV = 3.4; Non-teaching: CC = 0.5, VA = 1.9, VV = 0.7). *Conclusion*: VV ECMO had the highest median costs and payment among the ECMO types. Median payment was less than median hospitalization costs for all types of ECMO, apart from continuous VA ECMO.

## Introduction

Over the past two decades, there has been a significant increase in the use of extracorporeal membrane oxygenation (ECMO), particularly in the management of cardiogenic shock [1–7]. While ECMO has become an essential tool in various settings, including critical care, the operating room, extracorporeal life support (ECLS) transport, and procedure rooms, it remains a highly resource-intensive intervention with substantial associated costs and practice variation [8–10]. Outcomes and expenditures vary considerably depending on the indication, treatment center experience, case volume, and individual patient factors [4, 9, 10]. Given these complexities, there is a pressing need for a more comprehensive understanding of ECMO-related costs and payment to ensure its appropriate and sustainable use in healthcare systems.

## Materials and methods

This retrospective cohort analysis utilized the Medicare Fee-for-Service (FFS) database. The study population consisted of Medicare beneficiaries with continuous Medicare FFS coverage who experienced a hospitalization that included ECMO support between October 1, 2018, and December 31, 2022. To ensure a distinct episode analysis, only the first ECMO hospitalization per beneficiary within the study period was included.

### Endpoints

The primary endpoints of this study were as follows: (1) Total payment: the aggregate amount paid by Medicare FFS for the total resource utilization during the entire hospitalization. Total payment is the hospital-specific Medicare FFS payment, including Medicare outlier payment. (2) Estimated hospital costs: Hospital costs, which included all costs for the entire hospitalization, were calculated using cost-to-charge ratios (CCRs), which were determined using the total CCR calculated per Medicare provider from the CMS FY 2025, 2024, 2023, 2022, and 2021 Inpatient Prospective Payment System (IPPS) Final Rule Impact Files. CCRs were assigned to service dates, incorporating a 3-year lag to account for reporting delays. (3) Length of stay (LOS): LOS was defined as the total number of days from hospital admission to discharge. (4) Predominant diagnosis codes: Medicare Severity Diagnosis-Related Group (MS-DRG) and International Classification of Diseases, Tenth Revision (ICD-10), diagnosis codes were used to identify the primary reason for each hospitalization. Endpoints were stratified and analyzed by type of ECMO (i.e., continuous central [CC], venovenous [VV], venoarterial [VA]), determined using ICD-10 procedure codes; specific ICD-10 procedure codes related to continuous ECMO (Table 1); and discharge year. Discharge year was further stratified into three time periods because of changes made to ICD-10 diagnostic codes for ECMO: discharge (1) prior to October 1, 2018; (2) from October 1, 2018, through September 30, 2019; and (3) from October 1, 2019, through December 31, 2022. This manuscript reports endpoints from the most recent time period.

**Table 1:** ECMO ICD-10 Procedural Coding System (PCS) codes.

ICD-10 PCS Code	ECMO type	ICD-10 Code description
5A1522F	Continuous central	Extracorporeal or systemic assistance and performance physiological systems performance, circulatory continuous oxygenation membrane, central
5A15A2F	Intraoperative central	Extracorporeal or systemic assistance and performance physiological systems performance, circulatory intraoperative oxygenation membrane, central
5A1522G	Continuous VA	Extracorporeal or systemic assistance and performance physiological systems performance, circulatory continuous oxygenation membrane, peripheral venoarterial
5A1522H	Continuous VV	Extracorporeal or systemic assistance and performance physiological systems performance, circulatory continuous oxygenation membrane, peripheral venovenous
5A15A2G	Intraoperative VA	Extracorporeal or systemic assistance and performance physiological systems performance, circulatory intraoperative oxygenation membrane, peripheral venoarterial
5A15A2H	Intraoperative VV	Extracorporeal or systemic assistance and performance physiological systems performance, circulatory intraoperative oxygenation membrane, peripheral venovenous

### Statistical analysis

Continuous variables are reported as mean, standard deviation (SD), median, minimum, and maximum values, and categorical values are reported as frequencies (counts and percentages). Continuous variables were compared using t tests, and categorical variables were compared using chi-square tests. Statistical significance was defined as *p* < 0.05. Data analyses were performed using SAS Enterprise Guide 7.1 (SAS Institute Inc., Cary, NC, USA). All analyses were performed by one of the authors (A.R.).

## Results

There were 7295 hospitalizations that included ECMO support between October 1, 2019, and December 31, 2022. Among these, 4596 hospitalizations (63.0%) received payment for an amount less than the total claimed costs, and 2699 (37.0%) received a payment that exceeded the costs. In this analysis, we will refer to the 4596 patients whose hospitalization costs exceeded payment as the payment deficit cohort, and the 2699 patients whose hospitalization payment exceeded costs as the payment surplus cohort. A total of 1767 patients (65.5%) in the payment surplus cohort had an ICD-10 procedure code of 5A1522G (continuous peripheral VA ECMO) on the ECMO claims, 347 (12.9%) had code 5A1522F (continuous central VA or VV ECMO), and 611 (22.6%) had code 5A1522H (continuous peripheral VV ECMO). Less than 6% of patients in the payment surplus cohort had intraoperative ECMO codes (5A15A2F, 5A15A2G, or 5A15A2H) on the hospital claim.

Table 2 shows costs and payment for an entire ECMO hospitalization provided by FFS Medicare. Mean and median costs and payment were highest overall for hospitalizations involving intraoperative central ECMO. Among hospitalizations that included continuous ECMO support, mean and median costs and payment were highest for continuous VV ECMO. For both continuous and intraoperative ECMO, mean costs were higher than the mean payment. Median payment was greater than median costs for VA ECMO but less than median costs for all other types.

**Table 2:** Payments and costs for an entire ECMO hospitalization provided by FFS Medicare,^1^ discharge from October 1, 2019, through December 31, 2022.

	FFS Medicare (*N* = 7295)				
ICD-10 PCS Code^2^	Mean	SD	Median	Minimum	Maximum
5A1522F					
Payment	$292,203.88	$182,250.80	$236,062.31	$170.00	$1,917,188.15
Costs	$324,664.98	$253,440.10	$257,095.75	$11,346.36	$2,387,607.90
5A15A2F					
Payment	$345,280.92	$209,780.87	$302,384.01	$94,839.90	$933,241.98
Costs	$455,955.23	$311,980.98	$391,134.48	$67,491.89	$1,378,132.54
5A1522G					
Payment	$261,745.45	$170,144.69	$210,223.78	$129.05	$2,194,263.62
Costs	$273,458.91	$248,539.87	$206,452.51	$3,836.17	$3,244,288.53
5A1522H					
Payment	$304,238.74	$196,941.29	$244,208.79	$107.90	$1,917,188.15
Costs	$339,867.79	$283,126.56	$266,913.29	$11,505.87	$2,714,119.17
5A15A2G					
Payment	$165,500.84	$158,194.68	$116,405.88	$206.48	$1,541,738.29
Costs	$213,922.11	$200,913.41	$163,473.29	$12,890.30	$1,818,063.25
5A15A2H					
Payment	$152,968.37	$160,129.76	$98,030.18	$10,611.04	$971,568.06
Costs	$202,033.72	$216,521.56	$134,105.59	$10,714.05	$1,378,132.54

1 All dollars are adjusted to the January 2024 US dollar.

2 See Table 1 for definitions of ICD-10 PCS codes.

Patients are able to have more than >1 procedure code for ECMO per hospitalization.

**Figure 1:**
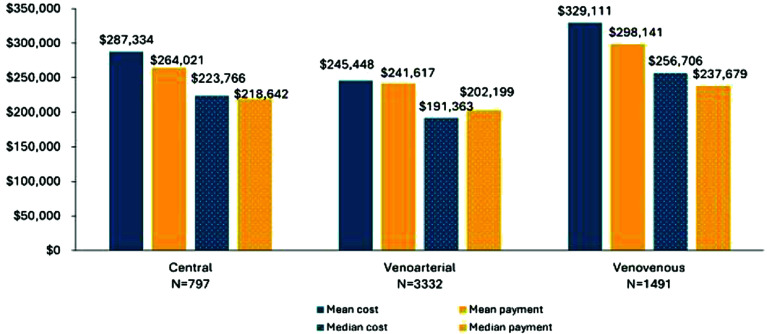
Mean and median cost and payment by type of continuous ECMO among those with DRG = 003 discharged between January 1, 2020, and December 31, 2022. The SDs for CC ECMO costs and payments were $224,261 and $157,882, respectively. The SDs for VA ECMO costs and payments were $212,822 and $145,456, respectively. The SDs for VV ECMO costs and payments were $285,745 and $198,160, respectively.

Figure 1 shows mean and median costs and payment for continuous ECMO hospitalizations with a DRG of 003 during a subset of the period defined in Table 2. Continuous VV ECMO had the highest average costs and Medicare FFS payment among the three types of continuous ECMO (CC, VA, and VV). Mean costs for continuous VV ECMO were $329,111, with a median of $256,706 and a standard deviation of $285,745. The mean payment was $298,141, with a median of $237,679 and a standard deviation of $198,16012. In comparison, continuous VA ECMO had mean costs of $245,448, a median of $191,363, and a standard deviation of $212,822. The mean payment for continuous VA ECMO was $241,617, with a median of $202,199 and a standard deviation of $145,45634. Continuous central ECMO had mean costs of $287,334, a median of $223,766, and a standard deviation of $224,261. The mean payment is $264,021, with a median of $218,642 and a standard deviation of $157,882.

Table 3 lists the 10 most common principal diagnoses (ICD-10) codes for the 2699 ECMO hospitalizations in the payment surplus cohort. The top 10 ICD-10 diagnosis codes account for 999 (37.0%) of these hospitalizations. The most frequent code was for non-ST elevation myocardial infarction, occurring in 215 hospitalizations (8.0%). COVID-19 was the third most frequent code, used in 105 (3.9%) of these hospitalizations. Sepsis due to unspecified and specified organisms was among the top 10 codes, used in 101 (3.7%) and 67 (2.5%) of the 2699 hospitalizations, respectively. Table 4 shows the 10 most common procedure codes in the payment surplus cohort.

**Table 3:** Top 10 principal diagnosis codes (ICD-10 Clinical Modification [CM] codes for payment surplus ECMO hospitalizations, October 1, 2019, through December 31, 2022).

		Frequency (%) at which payment > costs	
ICD-10 CM code	ICD-10 CM Code Description	All codes	Top 10 codes
		*N* = 2699	*N* = 949
I214	Non-ST elevation (NSTEMI) myocardial infarction	209 (7.7%)	209 (22.0%)
I350	Nonrheumatic aortic (valve) stenosis	106 (3.9%)	106 (11.2%)
U071	COVID-19	98 (3.6%)	98 (10.3%)
A419	Sepsis, unspecified organism	90 (3.3%)	90 (9.5%)
I2119	ST-elevation myocardial infarction involving other coronary artery of inferior wall	89 (3.3%)	89 (9.4%)
I130	Hypertensive heart and chronic kidney disease with heart failure and stage 1 through stage 4 chronic kidney disease, or unspecified chronic kidney disease	82 (3.0%)	82 (8.6%)
I2510	Atherosclerotic heart disease of native coronary artery without angina pectoris	82 (3.0%)	82 (8.6%)
I2109	ST-elevation myocardial infarction involving other coronary artery of anterior wall	74 (2.7%)	74 (7.8%)
A4189	Other specified sepsis	61 (2.3%)	61 (6.4%)
I340	Nonrheumatic mitral (valve) insufficiency	58 (2.1%)	58 (6.1%)

**Table 4:** Top 10 procedure codes in the patients’ index date bills in payment surplus hospitalizations.^*^

ICD-10 PCS Code	ICD-10 PCS Code description	Frequency (*N* = 2699)
		*n*
5A1522G	Extracorporeal or systemic assistance and performance physiological systems performance circulatory continuous oxygenation membrane, peripheral veno-arterial	1736
02HV33Z	Medical and surgical heart and great vessels insertion superior vena cava percutaneous infusion device no qualifier	938
0BH17EZ	Medical and surgical respiratory system insertion trachea via natural or artificial opening intraluminal device, endotracheal airway no qualifier	884
5A1221Z	Extracorporeal or systemic assistance and performance physiological systems performance cardiac continuous output no qualifier	882
5A1955Z	Extracorporeal or systemic assistance and performance physiological systems performance respiratory greater than 96 consecutive hours ventilation no qualifier	854
30233N1	Transfusion of nonautologous red blood cells into peripheral vein, percutaneous approach	829
03HY32Z	Medical and surgical upper arteries insertion upper artery percutaneous monitoring device no qualifier	703
5A1945Z	Extracorporeal or systemic assistance and performance physiological systems performance respiratory 24–96 consecutive hours ventilation no qualifier	696
B2111ZZ	Fluoroscopy of multiple coronary arteries using low osmolar contrast	696
5A1D90Z	Extracorporeal or systemic assistance and performance physiological systems performance urinary continuous, greater than 18 hours per day filtration no qualifier	635

* These codes were used in the payment surplus hospitalizations (*N* = 2699). Claims may include up to 25 codes. PCS indicates Procedural Coding System.

The mean age of the payment surplus cohort was 66.8 ± 12.0 years, compared with 65.2 ± 12.0 years for the payment deficit cohort (*p* < 0.0001). The patients in the payment surplus cohort were more frequently men (1657 of 2699, 61.4%) than women (1042 of 2699, 38.6%). Similarly, there were more men (2939 of 4596, 63.9%) than women (1657, 36.1%) in the payment deficit group.

Payment surplus hospitalizations were more frequent at teaching hospitals than nonteaching hospitals (2532 of 2699, 93.8% vs. 167 of 2699, 6.2%, respectively). Payment deficit hospitalizations were also more common at teaching hospitals than nonteaching hospitals (4356 of 4596, 94.8% vs. 240 of 4356, 5.2%, respectively). There was no difference between teaching and nonteaching status when comparing the two cohorts (*p* = 0.0828).

Payment surplus hospitalizations were more frequent in urban areas (1454 of 2698, 53.9%) than in rural centers (1244 of 2698, 46.1%). Conversely, payment deficit hospitalizations were more frequent in rural hospitals (2385 of 4595, 51.9%) than in urban hospitals (2210 of 4595, 48.1%). There was a statistically significant difference in the frequencies of rural and urban centers providing care when comparing the payment surplus and the payment deficit hospitalizations (*p* < 0.0001). As shown in Table 5, there were significant differences in the mean number of hospitalizations between urban and rural hospitals and between teaching and nonteaching hospitals.

**Table 5:** Mean number of hospitalizations for selected ECMO procedure codes by hospital setting and teaching status, October 1, 2019, through December 31, 2022.

	Hospital characteristic		
ICD-10 PCS Code	Rural (*N* = 221)	Urban (*N* = 403)	*p*-Value
5A1522F	2.4	1.4	0.0027
5A1522G	9.3	6.1	0.0054
5A1522H	4.9	2.2	<0.0001
	Nonteaching (*N* = 132)	Teaching (*N* = 494)	
5A1522F	0.5	2.1	<0.0001
5A1522G	1.9	8.6	<0.0001
5A1522H	0.7	3.8	<0.0001

LOS for payment surplus hospitalizations, both mean (14.8 ± 16.0 days) and median (10.0 days), were significantly shorter than LOS for payment deficit hospitalizations (mean = 42.5 ± 40.0 days; median = 31.0 days; *p* < 0.0001). In-hospital mortality was higher in patients with a payment surplus hospitalization than those with a payment deficit hospitalization (1812 of 2699, 67.14% vs. 1909 of 4596, 41.54%; *p* < 0.0001).

## Discussion

Similar to what has been reported previously [7, 9, 10], our data indicate significant variability in costs and Medicare FFS payment across the different ECMO types and highlight differences in resource usage among the ECMO types, with VV ECMO being the most resource-intensive.

Of the 2699 ECMO hospitalizations in the payment surplus cohort, the most frequent diagnosis code was for non-ST elevation myocardial infarction, occurring in 215 cases (8.0%). This is unsurprising given the notable increase in the use of ECMO, especially in cases of cardiogenic shock, over the past two decades [1–7]. Nonrheumatic aortic (valve) stenosis was second, reported in 107 cases (4.0%), and COVID-19 was the third most frequent diagnosis code, reported in 105 cases (3.9%). This finding is unsurprising given that the demand for ECMO surged as it became a critical intervention for severe COVID-19 cases during the peak of the pandemic in 2020 [11]. However, with the introduction of vaccines, the utilization of ECMO for COVID-19 decreased in subsequent years, particularly in 2021 and 2022, making COVID-19 less likely to be the top diagnosis for ECMO utilization. Sepsis due to unspecified and specified organisms was also among the top 10, used in 101 (3.7%) and 67 (2.5%) hospitalizations, respectively.

In payment surplus hospitalizations, the LOS was shorter. The shorter LOS correlated with a higher in-hospital mortality rate (67.1% in payment surplus hospitalizations vs. 41.5% in payment deficit hospitalizations), which may reflect the fact that patients who die during hospitalization often have reduced resource utilization due to a truncated clinical course. As a result, lower overall costs in these cases may contribute to the observed payment surplus. It should be noted that the data in the Medicare FFS database are from a primarily elderly population, which may have impacted this outcome. In an analysis of the impact of age on hospital LOS, patterns of patient disposition, and costs, Chung et al. [7] found that the odds of in-hospital mortality increased approximately 14% for every 10-year increase in age. They also similarly found that LOS was shortest and total median hospitalization costs were lower in the oldest group as compared with the youngest group.

Our research highlighted the differences in Medicare payment between rural and urban facilities, as well as teaching and non-teaching hospitals. These variations in Medicare payment between rural and urban hospitals are primarily influenced by the hospital wage index, which adjusts payments to account for geographic variation in labor costs. Urban areas generally have higher wage index values, resulting in higher payment rates for equivalent services compared to rural areas, where labor costs are typically lower. This adjustment promotes payment equity by aligning payment with local market conditions [12]. Additionally, teaching hospitals receive supplemental payments – known as indirect medical education adjustments – to offset the higher costs associated with training medical residents [13].

Discrepancies between hospital costs and Medicare payment for ECMO hospitalizations can occur in either direction, as shown in our payment surplus and payment deficit cohorts. ECMO patients are a complex population with wide variability in disease severity and LOS that would impact hospital-specific Medicare payment methodology. In some cases, Medicare payment may exceed hospital-reported costs. ECMO cases assigned to higher-paying DRGs, such as those that include mechanical ventilation or major complications or comorbidities, may result in payment that exceeds actual resource use if the patient’s clinical course is relatively uncomplicated [9, 14].

Conversely, it is also common for ECMO hospitalization costs to exceed Medicare payment amounts. ECMO is a highly resource-intensive therapy that often involves prolonged ICU stays, high staffing ratios, expensive technology, frequent laboratory testing, and management of complex complications such as bleeding, thrombosis, or multi-organ failure [15, 16]. Medicare’s prospective payment system does not always fully capture the variability in patient acuity or the intensity of services required, especially in patients requiring prolonged support or those who experience complications not reflected in coding or DRG assignment [17]. Furthermore, institutional variation in ECMO protocols, staffing models, and supply costs can contribute to wide variability in reported hospitalization costs [18].

ECMO patients are a heterogeneous group with substantial clinical variability. Indications range from short-term support for reversible respiratory failure to prolonged treatment for cardiogenic shock or as a bridge to transplant [19]. Resource needs vary significantly depending on the duration of support, clinical need, and patient-specific factors such as age, comorbidities, and complication profiles. This inherent variability challenges the alignment between fixed payment rates and individualized care needs.

As experience with ECMO continues to improve, we would expect improved outcomes and greater clarity on the factors contributing to the costs of treatment in ECMO.

### Limitations

The results in our research are based on the Medicare FFS database, which contains data on US patients only. The database does not include Medicare Advantage enrollees, Medicaid, or private payers. While Medicare FFS data provide valuable insights into Medicare FFS beneficiaries’ healthcare economics, it is insufficient for a comprehensive understanding of hospital ECMO economics. It is also important to evaluate data from various payers. The Medicare FFS inpatient database includes primarily patients aged ≥65 years, which may not be representative of the entire ECMO population. In addition, administrative databases may have potential bias due to confounding factors and missing data, which can affect overall findings. Finally, the Medicare data used in this analysis does not contain information on important variables relating to ECMO, including duration of cannulation. Future research, including how the duration of cannulation impacts costs and payment, is needed to better understand the economics of treating this complex population.

## Conclusion

This analysis demonstrated the variations in costs and Medicare FFS payment for hospitalizations that include different modes of continuous ECMO support. VV ECMO had the highest average costs and Medicare FFS payment among the three types of continuous ECMO. However, because of the large standard deviations around the mean values in this analysis, we think median values provide the best representation of the costs and Medicare FFS payment for hospitalizations that include ECMO. Median payment was less than median hospitalization costs in all types of ECMO, apart from continuous VA ECMO. Additional research is warranted to provide insight into these variations and to identify potential cost-saving strategies in the United States Medicare population.

## Data Availability

This retrospective analysis used data from the Medicare Fee-for-Service inpatient database and is governed under a data use agreement with the Centers for Medicare and Medicaid Services, which includes privacy and security requirements and data release policies. No new data were collected or analyzed in this study.

## References

[1] Gerke AK, Tang F, Cavanaugh JE, Doerschug KC, Polgreen PM. Increased trend in extracorporeal membrane oxygenation use by adults in the United States since 2007. BMC Res Notes. 2015;8:686. 26581610 10.1186/s13104-015-1678-7 PMC4650500

[2] Sauer CM, Yuh DD, Bonde P. Extracorporeal membrane oxygenation use has increased by 433% in adults in the United States from 2006 to 2011. ASAIO J. 2015;61:31–36. 25303799 10.1097/MAT.0000000000000160

[3] McCarthy FH, McDermott KM, Kini V, et al. Trends in U.S. extracorporeal membrane oxygenation use and outcomes: 2002–2012. Semin Thorac Cardiovasc Surg. 2015;27:81–88. 26686427 10.1053/j.semtcvs.2015.07.005 PMC4780346

[4] Strom JB, Zhao Y, Shen C, et al. National trends, predictors of use, and in-hospital outcomes in mechanical circulatory support for cardiogenic shock. EuroIntervention. 2018;13:e2152–e2159. 29400657 10.4244/EIJ-D-17-00947

[5] Thiagarajan RR, Barbaro RP, Rycus PT, et al. ELSO member centers. Extracorporeal Life Support Organization Registry International Report 2016. ASAIO J. 2017;63:60–67. 27984321 10.1097/MAT.0000000000000475

[6] Shah M, Patnaik S, Patel B, et al. Trends in mechanical circulatory support use and hospital mortality among patients with acute myocardial infarction and non-infarction related cardiogenic shock in the United States. Clin Res Cardiol. 2018;107:287–303. 29134345 10.1007/s00392-017-1182-2

[7] Chung M, Zhao Y, Strom JB, Shen C, Yeh RW. Extracorporeal membrane oxygenation use in cardiogenic shock: impact of age on in-hospital mortality, length of stay, and costs. Crit Care Med. 2019;47:e214–e221. 30585830 10.1097/CCM.0000000000003631 PMC6441360

[8] Harvey MJ, Gaies MG, Prosser LA. U.S. and international in-hospital costs of extracorporeal membrane oxygenation: a systematic review. Appl Health Econ Health Policy. 2015;13:341–357. 25894740 10.1007/s40258-015-0170-9

[9] Oude Lansinck-Hartgring A, van Minnen O, Vermeulen KM, van den Bergh WM, Dutch Extracorporeal Life Support Study Group. Hospital costs of extracorporeal membrane oxygenation in adults: a systematic review. PharmacoEconomics Open. 2021;5:613–623. 34060061 10.1007/s41669-021-00272-9 PMC8166371

[10] Mazzeffi M, Curley J, Gallo P, et al. Variation in hospitalization costs, charges, and lengths of hospital stay for coronavirus disease 2019 patients treated with venovenous extracorporeal membrane oxygenation in the United States: a cohort study. J Cardiothorac Vasc Anesth. 2023;37:1449–1455. 37127521 10.1053/j.jvca.2023.04.001 PMC10079589

[11] Milewski RC, Chatterjee S, Merritt-Genore H, et al. ECMO during COVID-19: a Society of Thoracic Surgeons/Extracorporeal Life Support Organization survey. Ann Thorac Surg Short Rep. 2023;1:168–173. 36545251 10.1016/j.atssr.2022.10.017 PMC9618293

[12] Centers for Medicare & Medicaid Services. Medicare program; hospital inpatient prospective payment systems for acute care hospitals and the long-term care hospital prospective payment system and final rule. Fed Regist. 2023;88(160):58934–59122. Available at: https://www.govinfo.gov/content/pkg/FR-2023-08-28/pdf/2023-16252.pdf. Accessed July 3, 2025.

[13] Centers for Medicare & Medicaid Services. Indirect Medical Education (IME). Available at: https://www.cms.gov/medicare/payment/prospective-payment-systems/acute-inpatient-pps/indirect-medical-education-ime. Updated April 2024. Accessed July 3, 2025.

[14] Centers for Medicare & Medicaid Services. FY 2024 IPPS Final Rule. Published August 2023. Available at: https://www.cms.gov/medicare/acute-inpatient-pps/fy-2024-ipps-final-rule-home-page. Accessed July 3, 2025.

[15] Barbaro RP, Odetola FO, Kidwell KM, et al. Association of hospital-level volume of extracorporeal membrane oxygenation cases and mortality: analysis of the Extracorporeal Life Support Organization registry. Am J Respir Crit Care Med. 2015;191:894–901. 25695688 10.1164/rccm.201409-1634OC PMC4435456

[16] Barrett KA, Hawkins N, Fan E. 2019. Economic evaluation of venovenous extracorporeal membrane oxygenation for severe acute respiratory distress syndrome. Crit Care Med. 47(2), 186–193. 10.1097/CCM.0000000000003465. 30312186

[17] Wunsch H, Linde-Zwirble WT, Angus DC, Hartman ME, Milbrandt EB, Kahn JM. The epidemiology of mechanical ventilation use in the United States. Crit Care Med. 2010;38:1947–1953. 20639743 10.1097/CCM.0b013e3181ef4460

[18] Schmidt M, Bailey M, Sheldrake J, et al. Predicting survival after extracorporeal membrane oxygenation for severe acute respiratory failure: the Respiratory Extracorporeal Membrane Oxygenation Survival Prediction (RESP) score. Am J Respir Crit Care Med. 2014;189:1374–1382. 24693864 10.1164/rccm.201311-2023OC

[19] Brodie D, Bacchetta M. Extracorporeal membrane oxygenation for ARDS in adults. N Engl J Med. 2011;365(20):1905–1914. 22087681 10.1056/NEJMct1103720

